# Propofol Induces Apoptosis of Neurons but Not Astrocytes, Oligodendrocytes, or Neural Stem Cells in the Neonatal Mouse Hippocampus

**DOI:** 10.3390/brainsci7100130

**Published:** 2017-10-14

**Authors:** Yasheng Yan, Shigang Qiao, Chika Kikuchi, Ivan Zaja, Sarah Logan, Congshan Jiang, Thiago Arzua, Xiaowen Bai

**Affiliations:** 1 Department of Anesthesiology, Medical College of Wisconsin, Milwaukee, WI 53226, USA; yashengyan@mcw.edu (Y.Y.); qiaoshigang@163.com (S.Q.); usakop10@gmail.com (C.K.); ivan.zaja@yahoo.com (I.Z.); cjiang@mcw.edu (C.J.); 2 Department of Physiology, Medical College of Wisconsin, Milwaukee, WI 53226, USA; sarahlogan@mcw.edu (S.L.); tarzua@mcw.edu (T.A.); 3 Department of Biochemistry and Molecular Biology, School of Basic Medical Science, Xi’an Jiaotong University Health Science Center, Xi’an 710061, Shaanxi, China

**Keywords:** propofol, apoptosis, neurons, astrocytes, oligodendrocytes, neural stem cells, selective vulnerability

## Abstract

It has been shown that propofol can induce widespread apoptosis in neonatal mouse brains followed by long-term cognitive dysfunction. However, selective brain area and cell vulnerability to propofol remains unknown. This study was aimed to dissect toxic effect of propofol on multiple brain cells, including neurons, astrocytes, oligodendrocytes, and neural stem cells (NSCs). Seven-day-old mice were intraperitoneally administrated propofol or intralipid as a vehicle control for 6 hours. To identify vulnerable cells undergoing apoptosis following propofol exposure, brain sagittal sections were co-stained with antibodies against an apoptosis marker along with neuron, astrocyte, oligodendrocyte, or NSC markers using immunofluorescence staining. The results showed widespread apoptosis in propofol-treated brains (apoptotic cells: 1.55 ± 0.04% and 0.06 ± 0.01% in propofol group and intralipid-treated control group, respectively). Apoptotic cell distribution exhibits region- and cell-specific patterns. Several brain regions (e.g., cerebral cortex and hippocampus) were more vulnerable to propofol than other brain regions. Most apoptotic cells in the hippocampus were located in the cornus ammonis 1 (CA1) subfield. These apoptotic cells were only detected in neurons and not astrocytes, oligodendrocytes, or NSCs. These data demonstrate that different brain regions, subfields, and different types of neuronal cells in mice exhibit various vulnerabilities to propofol. Understanding region- and cell-specific susceptibility to propofol will help to better understand cellular contribution to developmental neurotoxicity and further develop novel therapeutic targets.

## 1. Introduction

Propofol, a short-acting agent with rapid induction and recovery times, is one of the most widely used general intravenous anesthetics in pediatrics and obstetrics [1]. Growing evidence suggests that exposure of propofol at clinically relevant doses and durations to developing animals (including both rodents and primates) during brain growth spurt causes widespread neuronal cell death followed by long-term memory and learning abnormalities, as observed in other anesthetic (e.g., isoflurane, ketamine, and sevoflurane) neurotoxicity studies [2,3,4,5,6,7]. This brain growth spurt period ranges differently among species. For rodents, this period occurs during the first two weeks after birth. In rhesus monkeys, it starts from approximately 115-day gestation (G) and continues to postnatal day (P) 60, while in humans, it ranges from about the third trimester of pregnancy up to approximately the third year after birth [7,8]. For instance, seven-day-old mice received one intraperitoneal injection of propofol (150 mg/kg), which produced persistent anesthesia for 6 h, and significantly increased activated caspase 3 (apoptotic marker) levels in the cortex and hippocampus [3]. In another multiple propofol exposure study on seven-day-old Sprague-Dawley rats with intraperitoneal injection of propofol at 50, 100, and 150 mg/kg/day for five consecutive days, no obvious apoptotic effect was observed at a low dose (50 mg/kg), whereas the 100 mg/kg or 150 mg/kg dose increased apoptotic factors (caspase 3 and Bax) levels [2]. The neurotoxicity effect was also observed in developing non-human primate (NHP) rhesus macaque brains [4].

However, the vulnerability of different brain cells to anesthetics in different species remains largely unknown. So far, there is only one report describing the vulnerability of different brain cells. In this study, fetal or neonatal macaques (G120 or P6, respectively) were exposed to propofol for 5 h or to no anesthesia. Propofol exposure caused a significant increase of neuron and oligodendrocyte apoptosis 3 h after exposure [4]. The similar pathological changes were also observed in isoflurane-treated macaque brains [9]. However, which types of cells in neonatal mice are more vulnerable to propofol remains unknown. The central nervous system (CNS) is composed of two major cell types: neuron and glia (astrocytes and oligodendrocytes). Neural stem cells (NSCs) have the ability to proliferate and differentiate into neurons, astrocytes, and oligodendrocytes, and play important roles in brain development. The apoptotic effect of propofol on NSCs in the developing animal has not been reported. Thus, the aim of this current study was to identify developing mouse brain areas vulnerable to developmental propofol exposure, and the effect on their constituent neuronal cells, including neurons, astrocytes, oligodendrocytes, and NSCs.

## 2. Materials and Methods

### 2.1. Animals

All animal experiments (project identification code: AUA00001767) were approved by the Institutional Animal Care and Use Committee at the Medical College of Wisconsin on 8/19/2010. Breeding pairs of male and female C57BL/6 mice (Jackson Laboratory, Bar Harbor, ME, USA) were housed in a 12/12-h light-dark cycle at 22 °C with free access to food and water. The greatest vulnerability of the developing brain to anesthetics occurs during the brain growth spurt period. In mice, brain growth peaks at approximately seven days after birth [10]. Thus, seven-day-old mice were used in all experiments, and randomly divided into control and propofol treatment groups.

### 2.2. Propofol Administration

Seven-day-old mice were placed in a temperature-controlled incubator (37 °C) and intraperitoneally received propofol (ZOETIS, Parsippany, NJ, USA) or 10% intralipid (Fresenius Kabi AB, Uppsala, Sweden) as a vehicle control. The administered propofol was a total of four injections of 50 mg/kg body weight at 90 min interval. This dose of propofol was selected based on the previous reports by other groups showing that the dosage required to induce a surgical plane of anesthesia in mice was 200 mg/kg and that sub-anesthetic doses of 50 and 100 mg/kg propofol could trigger neuroapoptosis [5]. In our pilot experiments, we observed a similar anesthetic depth conferred by 50 mg/kg propofol as evidenced by the loss of righting reflex but a remaining response to toe pinch. One injection of propofol maintained anesthesia in mice for approximately 90 min [5]. Therefore, propofol was administered every 90 min throughout the experiments for a total of four times to maintain 6-h anesthesia. Immediately after propofol exposure, brain tissues were harvested for Western blot and immunofluorescence staining analysis as described below.

### 2.3. Identification of Vulnerable Cellular Types Following Propofol Exposure Using Immunofluorescence Staining

Mice were subjected to perfusion-fixation using 10% zinc formalin fixation solution (Richard-Allan Scientific, San Diego, CA, USA) through the left ventricle once the animal has reached a surgical plane of anesthesia with isoflurane. Afterward, the brains were quickly removed and further post-fixed with formalin overnight at room temperature. The brain tissues were cut into the right and left hemispheres along the anatomic midline. Paraffin-embedded brain hemisphere tissue blocks were then cut into 4 µm-thick sagittal sections. The cut position was around 600 µm distance to the midline. The sections were deparaffinized with xylenes, hydrated through graded ethanol, and then subjected to antigen retrieval by incubation in target retrieval solution (Dako, Santa Clara, CA, USA) for 20 min in a boiling steamer. Then, sections were washed three times with phosphate buffered saline (PBS) containing 0.5% Triton X-100 (Sigma, Milwaukee, WI, USA), and blocked with 10% donkey serum (Thermo Fisher Scientific, Waltham, MA, USA) for 30 min at room temperature. In order to identify which types of neuronal cells undergo apoptosis following propofol exposure, the sections were co-stained with the following primary antibodies: rabbit anti-activated caspase 3 (apoptosis marker; Cell Signaling) along with either mouse anti-neuronal nuclear antigen (NeuN: neuron marker; Millipore, Billerica, MA, USA), goat anti-glial fibrillary acidic protein (GFAP: astrocyte marker; Abcam, Cambridge, MA, USA), mouse anti-myelin basic protein (MBP: oligodendrocyte marker; Santa Cruz, Dallas, Texas, USA), or mouse anti-nestin (NSC marker; Millipore, Billerica, MA, USA) in a humidified chamber for 1 h at 37 °C. After three washes, slides were incubated with Alexa Fluor 488-conjugated donkey anti-mouse IgG or goat IgG, together with Alexa Fluor 594-conjugated donkey anti-rabbit (Thermo Fisher Scientific, Waltham, MA, USA) for 45 min at 37 °C. After three more washes with PBS, the slides were stained for nuclei with Hoechst 33342 (Thermo Fisher Scientific, Waltham, MA, USA). The coverslips were mounted on glass slides with mounting medium (Thermo Fisher Scientific, Waltham, MA, USA) and sealed with nail polish. The sections of hemispheres were photographed using an Olympus Fluorescent Slide Scanner and OlyVIA2.4 software (Olympus Corporation of the Americas, Center Valley, PA, USA). Total apoptotic cells were calculated as a percentage according to the following formula: activated caspase 3–positive cells/the number of total cell nuclei in brain sections.

### 2.4. Quantification of Apoptosis Using Western Blot

Western blot assay was performed as described previously [11]. Brieflly, following exposure to propofol or intralipid, brain tissues were collected and lysed and sonicated in RIPA lysis buffer (Cell Signaling, Danvers, MA, USA) containing a phosphatase and protease inhibitor cocktail (Roche Diagnostics, Indianapolis, IN, USA). Lysates were centrifuged at 10,000× *g* for 10 min at 4 °C. Total protein concentration of the supernatants was determined using a DC Protein Assay Reagents Package kit (Bio-Rad, Hercules, CA, USA). The samples were boiled for 5 min at 97 °C. Twenty five μg of protein was loaded per lane for sodium dodecyl sulfate polyacrylamide gel electrophoresis gel separation, and then transferred to nitrocellulose membrane. Membranes were blocked with blocking buffer (Thermo Fisher Scientific, Waltham, MA, USA) and incubated overnight at 4 °C with the following primary antibodies: rabbit anti-activated caspase 3 (Cell Signaling, Danvers, MA, USA) and rabbit anti-actin (internal control; Santa Cruz, Dallas, Texas, USA). The primary antibodies were then washed out with Tris-buffered saline with 0.1% Tween 20 buffer. Subsequently, the membranes were incubated with secondary antibodies conjugated to horseradish peroxidase (Cell Signaling, Danvers, MA, USA) for 1 h at room temperature, and labeled proteins were detected with chemiluminescence detection reagent (Cell Signaling, Danvers, MA, USA) and obtained on X-ray film. Optical densities of activated caspase 3 and actin were quantified using ImageJ 1.47v software (Wayne Rasband, National Institutes of Health). The activated caspase 3 abundance was normalized to actin and reported as a percentage of the intralipid vehicle control.

### 2.5. Counting Apoptotic Neuronal Cells in Hippocampus

Our pilot study showed that several brain regions (e.g., cerebral cortex, caudate/putamen, and hippocampus) were more vulnerable to anesthetics than other regions. Since hippocampus is a brain region primarily associated with memory and learning, and our pilot experiments indicated that most of the apoptotic cells were observed in the cornus ammonis 1 (CA1) subfield of the hippocampus, we confined the quantitative assessment of vulnerable neuronal cells to CA1 following immunofluorescence staining as described above. The apoptotic cells were counted in a blinded manner. The percentage of individual types of neuronal cells which underwent apoptosis was expressed as percentage of total apoptotic cells based on the formula: number of specific neuronal cells with apoptosis/number of total activated caspase 3-potitive apoptotic cells × 100. Apoptotic neurons, astrocytes, oligodendrocytes, and NSCs were defined as activated caspase 3/NeuN, activated caspase 3/GFAP, activated caspase 3/MBP, or activated caspase 3/nestin double-positive cells, respectively.

### 2.6. Statistical Analysis

All values described were expressed as the mean ± standard error of the mean. All experimental data were obtained from three mice per group. The statistically significant differences of the data between control and propofol treatment groups were analyzed by unpaired Student’s *t*-test using the SigmaPlot 12.5 software (Systat Software, Inc., San Jose, CA). A level of *p* < 0.05 was considered to be statistically significant.

## 3. Results

### 3.1. Propofol Exposure Induces Widespread Apoptosis in Neonatal Mouse Brains in a Region-Dependent Manner

**Figure 1:**
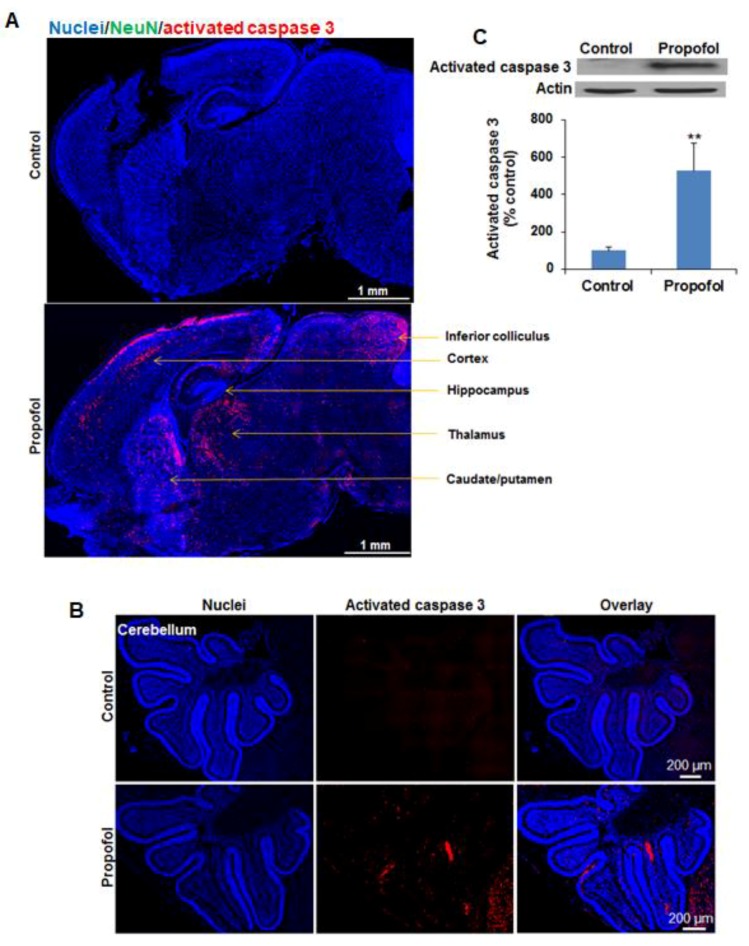
Propofol exposure induces widespread apoptotic cell death in neonatal mouse brains in a region-specific manner. Seven-day-old mice intraperitoneally received propofol (50 mg/kg body weight) or 10% intralipid as a vehicle control for 6 h. (**A**,**B**) The fluorescence images of immunostained sagittal section of mouse brains show that several brain regions such as cerebral cortex, caudate/putamen, thalamus, hippocampus, subiculum, entorhinal cortex, inferior colliculus, and cerebellum are more vulnerable to propofol than other regions as evidenced by the higher density of apoptotic cells observed in these regions. Blue are cell nuclei stained with Hoechst 33343 and red are activated caspase 3–positive apoptotic cells; (**C**) Western blot analysis demonstrated that the activated caspase 3 level was significantly increased in the propofol-treated brains (527.68 ± 146.39% control group). Scale bars = 1 mm (**A**) and 200 µm (**B**); ** *p* < 0.01.

A subanesthetic dose of propofol exposure for 6 h induced widespread apoptosis in seven-day-old mouse brains (Figure 1A,B). Based on the evaluation of the density and distribution of activated caspase 3–positive apoptotic signals, we found that several brain regions such as the cerebral cortex, caudate/putamen, thalamus, hippocampus, subiculum and entorhinal cortex, inferior colliculus, and cerebellum, were more vulnerable to propofol than other regions. Western blot analysis further confirmed the observation from immunofluorescence staining. The activated caspase 3 level was significantly increased in the propofol-treated brains (527.68 ± 146.39% control group; *n* = 3; *p* < 0.05 (Figure 1C). Apoptotic cells were 1.55 ± 0.04% and 0.06 ± 0.01% of total cells in mouse brain sections in propofol and control groups, respectively (*n* = 3; *p* < 0.05).

### 3.2. Different Subfields in Several Brain Regions Exhibit Varying Vulnerability to Propofol

**Figure 2:**
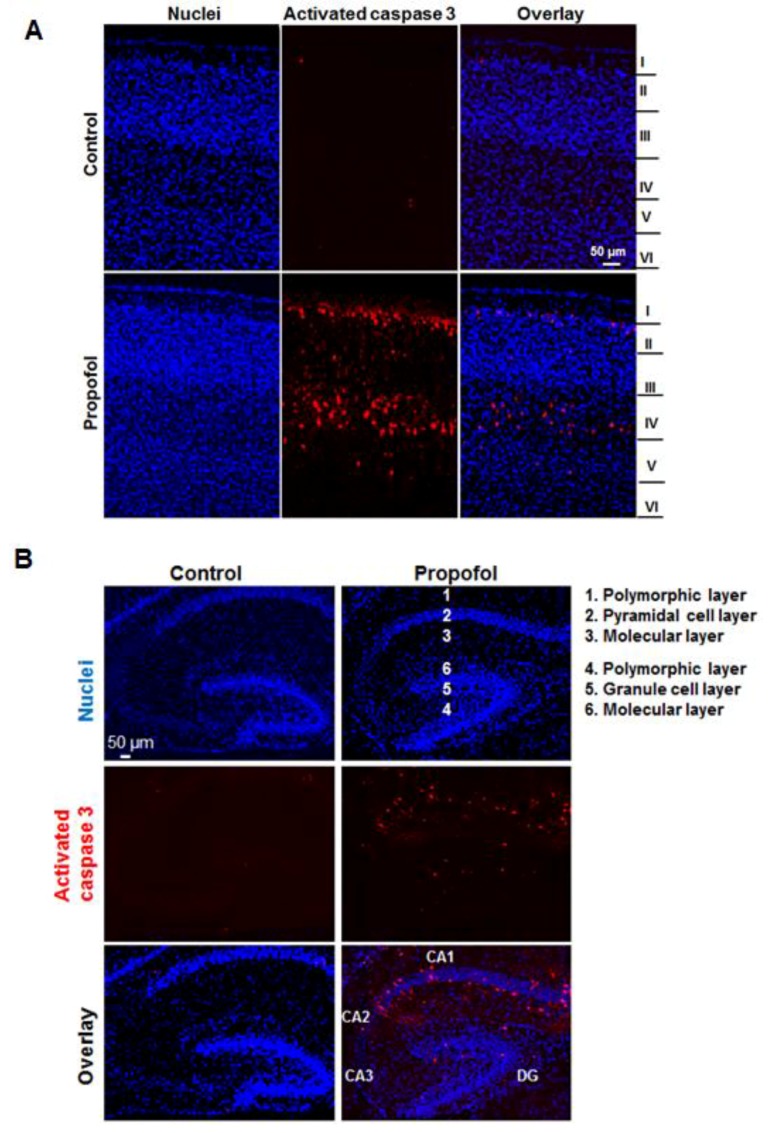
Different subfields in several brain regions exhibit various vulnerability to propofol. Fluorescence images of the cortex (**A**) and hippocampus (**B**) showed that obvious apoptotic cells were observed in layers II to V of cortex, and in cornus ammonis 1 (CA1) and dentate gyrus (DG) subfields of the hippocampus treated with propofol. The numbers represent three distinct layers in both CA1 and DG. Blue are cell nuclei and red are activated caspase 3–positive apoptotic cells. Scale bar = 50 µm.

Apoptotic cells in several brain regions such as the cortex and hippocampus were not evenly distributed. For example, obvious apoptotic cells were observed in specific layers (layers II to V in cortex) and most apoptotic cells were located in layers II and IV (Figure 2A). The hippocampus proper is composed of four subfields: dentate gyrus (DG) and CA fields (CA1-3). Both DG and CA subfields are made up of three prominent layers. In the hippocampus, most of apoptotic cells were located in the pyramidal cell layer of CA1 and granule cell layer of DG (Figure 2B).

### 3.3. Propofol Induces Apoptosis in Neurons in Hippocampus

**Figure 3:**
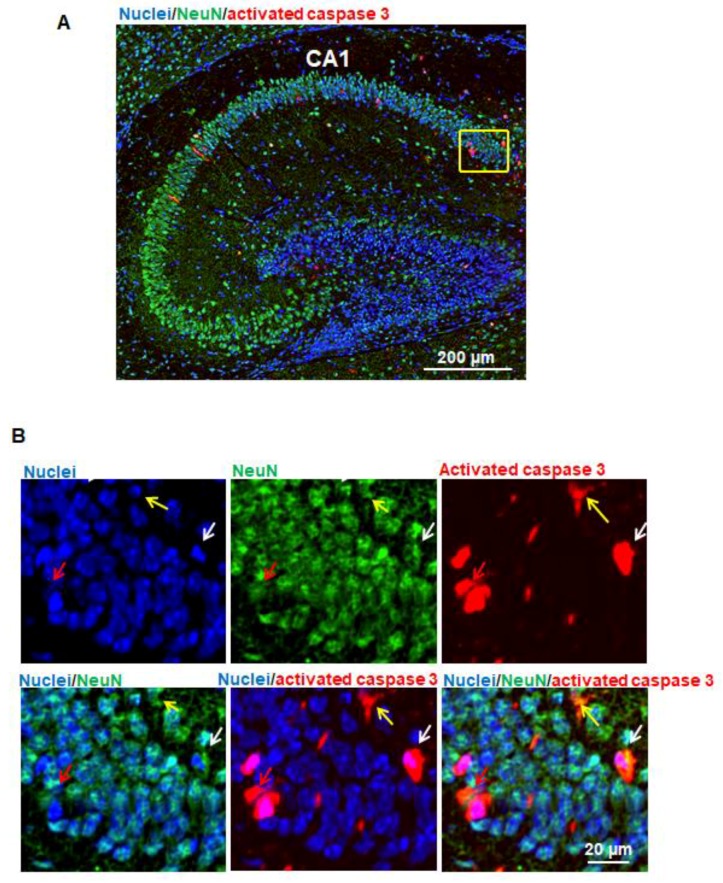
Propofol induces apoptosis in neurons in the hippocampus. (**A**) Fluorescence images of the hippocampus treated with propofol. In order to identify whether neurons in hippocampus undergo apoptosis following propofol exposure, the brain sagittal section were co-stained with activated caspase 3 and neuronal nuclear antigen (NeuN; neuron marker). Cell nuclei were stained with Hoechst 33342. Blue, green, and red represent cell nuclei, NeuN, and activated caspase 3 signals. Scale bar = 200 µm; (**B**) The magnified view of the yellow-boxed region shown in Figure 3A. These images include the individual channel and overlaid images and show that some activated caspase 3 and NeuN signals were co-localized in the same cells, suggesting that propofol induces apoptotic neuron death. Three representative apoptotic neurons are indicated by yellow, white, and red arrows. Scale bar = 20 µm.

In order to identify whether neurons in the hippocampus undergo apoptosis following propofol exposure, the brain sagittal sections were subject to immunofluorescence staining for visualizing the signals of apoptotic cells (red; activated caspase 3), neurons (green; NeuN), and cell nuclei (blue). Most of NeuN-positive neurons were located in the pyramidal cell layer of hippocampus (Figure 3A). Some activated caspase 3–positive apoptotic signals and NeuN-positive neuron signals were co-localized in the same cells, suggesting that propofol induces apoptotic neuron death (Figure 3B). Since most of apoptotic cells were located in CA1 subfield of the hippocampus, we counted number of apoptotic neurons and found that apoptotic neurons represent 50.67 ± 2.62% (*n* = 3) total apoptotic cells observed in CA1.

### 3.4. Propofol Does Not Trigger Astrocyte Apoptosis in Hippocampus

**Figure 4:**
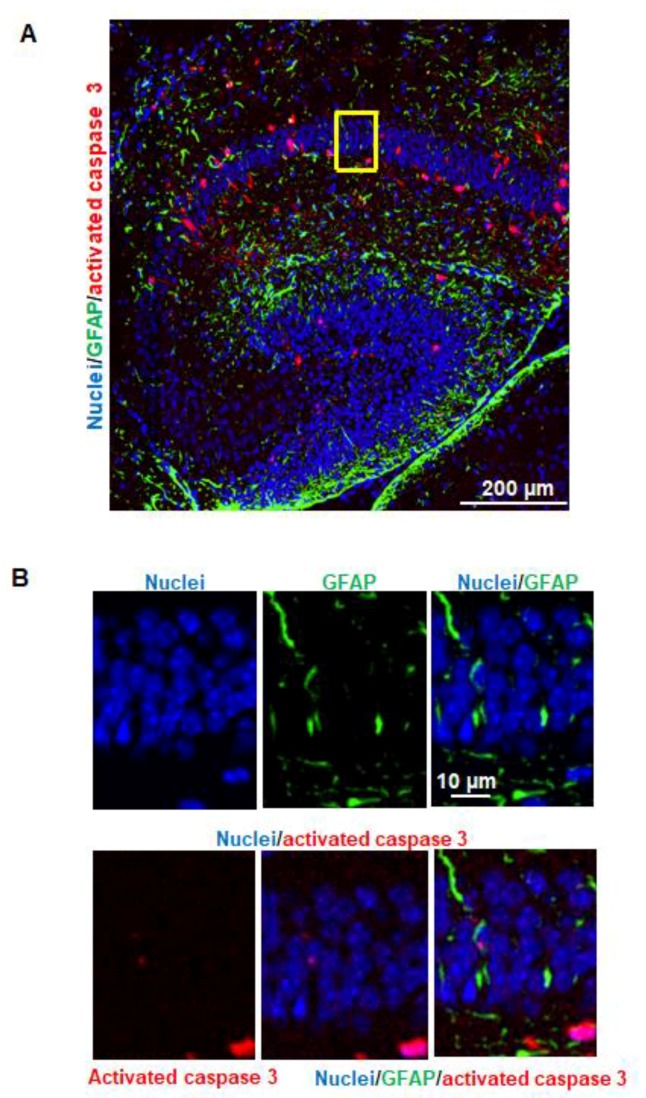
Propofol does not trigger astrocyte apoptosis in the hippocampus. (**A**) Fluorescence images of the hippocampus treated with propofol. The brain sagittal sections were co-stained with activated caspase 3 and glial fibrillary acidic protein (GFAP; astrocyte marker). Cell nuclei were stained with Hoechst 33342. Blue, green, and red represent cell nuclei, GFAP, and activated caspase 3 signals, respectively. Scale bar = 200 µm; (**B**) The magnified view of the yellow-boxed region shown in Figure 4A. These images include the individual channel and overlaid images and show that activated caspase 3 and GFAP signals were not co-localized in the same cells, indicating that propofol does not cause astrocyte apoptosis. Scale bar = 10 µm.

The brain sagittal sections were subject to immunostaining for detection of 3 different signals: activated caspase 3 (red), GFAP (green), and cells nuclei (blue). The majority of GFAP–positive astrocytes were distributed in the polymorphic and molecular layers (Figure 4A). The activated caspase 3 and GFAP signals were not co-localized in the same cells (Figure 4B), suggesting that propofol does not induce apoptotic astrocyte death.

### 3.5. Propofol Does Not Trigger Apoptosis in Oligodendrocytes in Hippocampus and Other Brain Regions

**Figure 5:**
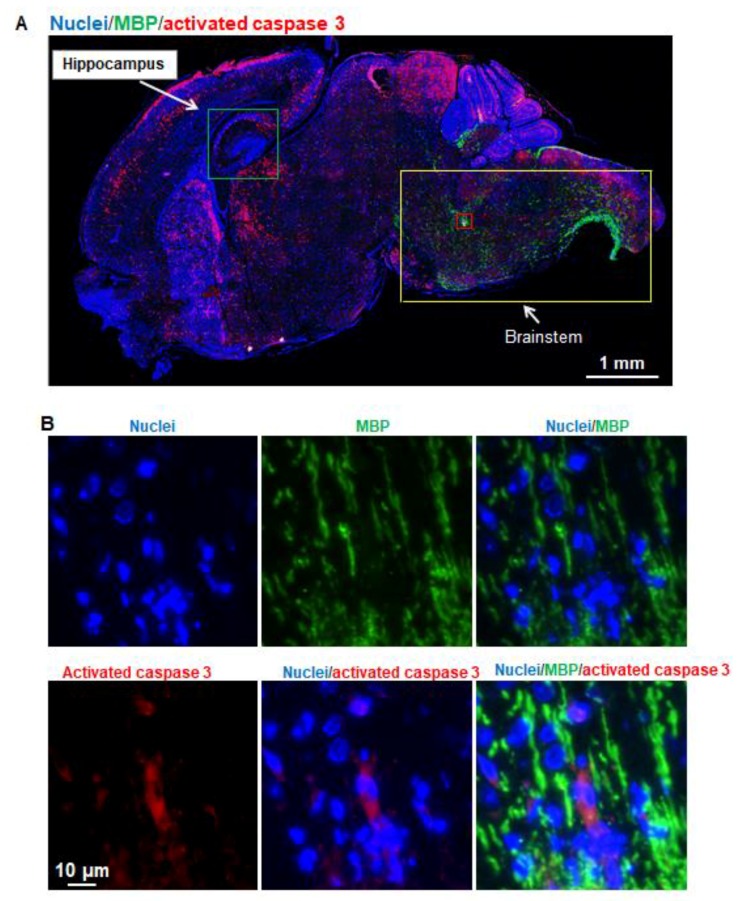
Propofol does not trigger apoptosis in oligodendrocytes in the hippocampus. In all images of Figure 5, Blue, green, and red represent cell nuclei, myelin basic protein (MBP; oligodendrocyte marker), and activated caspase 3 signals, respectively. (**A**) Fluorescent images of sagittal section of mouse brains treated with propofol. MBP were barely detected in hippocampus marked by green box and other brain regions with severe apoptosis (e.g., cortex). Compared with the MBP and apoptotic signals in vulnerable brain regions, stronger and more MBP signals were observed in brainstem including midbrain, pons, and medulla regions marked in the yellow-boxed view, while fewer activated caspase 3 signals were detected in these regions, suggesting that brain regions with advanced myelination are not vulnerable to propofol; (**B**) The magnified view of the red-boxed region shown in Figure 5A. These images include the individual channel and overlaid images, and show that activated caspase 3 and MBP signals were not co-localized in the same cells, suggesting that propofol does not induce oligodendrocyte apoptosis. Scale bars = 1 mm (**A**) or 10 µm (**B**).

The brain sagittal sections were subject to immunofluorescence staining for visualizing the signals of apoptotic cells (red; activated caspase 3), oligodendrocytes (green; MBP), and cell nuclei (blue). We only found very few MBP signals in the magnified hippocampus images (data not shown). The MBP and activated caspase 3 double-positive cells were not observed, suggesting that (1) there was very little myelination in the seven-day-old mouse hippocampus, and (2) propofol does not induce oligodendrocyte apoptosis in hippocampus. Since the previously published rhesus macaque studies showed that both isoflurane and propofol induced oligodendrocyte apoptosis [4,9], we then analyzed apoptosis and oligodendrocyte signals in different brain regions. We also barely found the MBP-positive oligodendrocyte signals in the vulnerable regions such as caudate/putamen, thalamus, and cortex where there were more apoptotic cells observed following propofol exposure. However, strong and major MBP signals were observed in brainstem regions including midbrain, pons, and medulla where only fewer apoptotic cells were detected (Figure 5A). In addition, the activated caspase 3 and MBP signals were not co-localized in the same cells (Figure 5B), indicating that propofol does not cause oligodendrocyte apoptosis and the brain regions with advanced myelination are not vulnerable to propofol.

### 3.6. Propofol Does Not Induce Apoptosis in NSCs in Hippocampus

**Figure 6:**
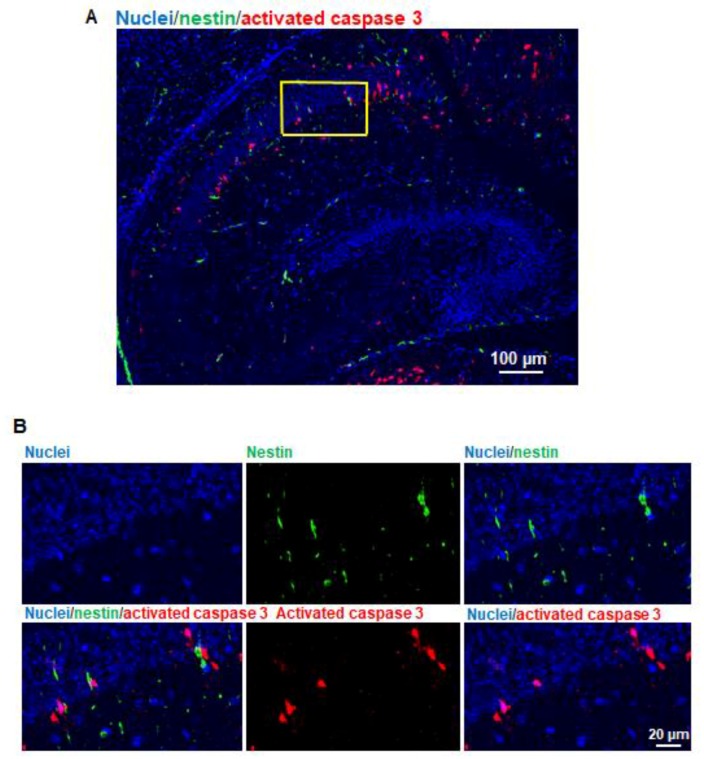
Propofol does not induce apoptosis in neural stem cells (NSCs) in hippocampus. (**A**) Fluorescence images of hippocampus treated with propofol. The brain sagittal sections were co-stained with activated caspase 3 and nestin (NSC marker). Cell nuclei were stained with Hoechst 33342. Blue, green, and red represent cell nuclei, nestin, and activated caspase 3 signals, respectively. Scale bar = 100 µm; (**B**) The magnified view of the yellow-boxed region marked in Figure 6A. These images include the individual channel and overlaid images and show that activated caspase 3 and nestin signals were not co-localized in the same cells, suggesting that propofol does not induce the apoptotic NSC death. Scale bar = 20 µm.

The brain sagittal sections were co-stained with activated caspase 3 and nestin (NSC marker) (Figure 6A). The activated caspase 3 (red) and nestin (green) signals were not co-localized in the same cells in the hippocampus (Figure 6B), suggesting that propofol does not induce apoptotic NSC death.

## 4. Discussion

The aim of the current study was to focus on the dissection of the apoptotic effect of propofol on seven-day-old mice brains. Specifically, for the first time, we investigated the vulnerability of different brain regions and different types of neuronal cells to propofol exposure. The major findings of this study are (1) propofol induces widespread cell apoptosis in the neonatal mouse brain, (2) different brain regions, subfields and different types of neuronal cells exhibit various vulnerability to propofol, and (3) propofol induces apoptosis of neurons, but not astrocytes, oligodendrocytes, or NSCs.

Apoptosis is a commonly recognized side effect of anesthetics in developing animals [12,13,14,15]. However, there are no reports regarding the distribution of apoptotic cells in different brain regions in mice following propofol exposure. Our data showed that more apoptotic cells were observed in several brain regions including cerebral cortex, caudate/putamen, hippocampus, subiculum, entorhinal cortex, thalamus, inferior colliculus, and cerebellum, while fewer apoptotic cells were found in the hypothalamus, superior colliculus, and brainstem (midbrain, pons, and medullas). The effect of propofol (5 h) on the NHP (rhesus macaque) fetus (G120, full term = 165 days) resulted in a similar apoptosis in the cerebral cortex, caudate/putamen, thalamus, inferior colliculus, and cerebellum with the notable exception of the hippocampus, which showed very little effect [4]. Other anesthetic drugs (e.g., isoflurane and ketamine) also did not cause obvious apoptosis in the hippocampus of NHP [7,9]. The lack of damage to the hippocampus in NHP was inconsistent with our results ( Figure 1A and Figure 2B) and previous reports regarding propofol and other anesthetic drug (isoflurane, ketamine, and sevoflurane) toxicity on mice [3,16,17]. There might be several explanations for the difference in vulnerability to anesthetics between the developing hippocampi of NHP and mice: (1) the vulnerability of neuronal cells from different species might differ due to different internal signaling of neuronal cells and developmental events (e.g., oligodendrocyte differentiation, and myelination) and different response to external anesthetic stress; (2) the doses and exposure duration of propofol used in the NHP study (anesthetic dose, 5 h) and our current mouse study (a subanesthetic dose, 6 h) were not same, which might contribute to the different apoptotic effect; (3) although it has been reported that a fetal age of G120 corresponds in brain age to that of a late third-trimester human fetus and around P2–7 in rodents [4,18], the exact equivalent developmental age across different species is still not clear. Thus, future studies will include mice with different developing ages in order to dissect the vulnerability window of the hippocampus to anesthetics.

Our experimental results also showed that propofol caused severe neuronal damage to the selectively vulnerable areas of the brain and to selectively vulnerable subfields of respective regions. In the cortex, the apoptotic signals were located in specific layers (layers II to V in cortex) (Figure 2A). This distinct laminar-like distribution of apoptosis was also observed in NHP brains treated with propofol and isoflurane [4,7,9]. In the hippocampus, the vulnerable area majorly encompassed the CA1 subfield (Figure 2B). Since anesthetics induce abnormal learning and memory disabilities, and the hippocampus is primarily associated with memory and spatial navigation, we focused on the dissection of the vulnerability of cell types in CA1 area. We found that propofol induced the apoptosis in neurons (Figure 3) but not astrocytes (Figure 4), oligodendrocytes (Figure 5), or NSCs (Figure 6). However, apoptotic NeuN-positive neurons only represent 50.67 ± 2.62% total apoptotic cells observed in the CA1. The majority of apoptotic neurons were located in the pyramid cell layers (Figure 3). Remaining non-NeuN-positive apoptotic cells in CA1 might include (1) other types of brain cells that were not analyzed in the current study such as microglial cells and vascular cells and (2) neurons that may lose NeuN signals at a later stage of the apoptosis.

The vulnerability of the CA1 subfield was also observed in several other neurodegenerative diseases, such as Alzheimer’s disease and ischemic stroke [19,20]. The potential mechanisms underlying selective vulnerability in neurodegeneration are complex, multifactorial, and incompletely understood. For our study, the vulnerability of CA1 area might be related to immature status of neurons. In immature neurons, activation of γ-aminobutyric acid types A receptor (GABAAR) induces a calcium influx. It is commonly accepted that propofol can potentiate GABAAR and subsequently cause excitatory neurotoxicity in immature neurons [21]. In addition, there are differences in the energy demand of different neurons. CA1 neurons are characterized by particularly high energy consumption [22]. The high energy demand may increase the susceptibility of CA1 neurons to propofol insults.

Astrocytes, the largest population of glial cells, exert a number of important functions (e.g., providing metabolic and physical support to other neural cells and regulating synaptogenesis and myelination) during brain development. We did not detect apoptotic astrocytes (Figure 4). This data is consistent with the observation of the NHP study [4]. Oligodendrocytes are a type of large glial cells in the CNS. The main function of oligodendrocytes is to provide support and insulation to neuronal axons of neurons by creating the myelin sheath, the lipid-enriched axon-coating membrane. Before myelination is initiated, oligodendrocyte precursors transform first into pre-myelinating oligodendrocytes and then into mature myelin-producing cells. MBP is a major structural component of the myelin sheath and is localized to the cytoplasmic surface of the plasma membrane and myelin membrane. In mice, myelination occurs in a conserved and region-dependent pattern in brains, with synthesis beginning in the brainstem and progressing over the other brain regions during several weeks. It starts at birth and peaks at approximately P20. Myelination is achieved in almost all regions in brain around 45–60 days postnatally [23].

In the current study, we found that (1) oligodendrocytes were barely detected in the brain regions where brain damage caused by apoptosis were most severe and (2) the higher density of MBP-positive oligodendrocytes were observed in brainstem regions in seven-day-old mice, and those regions with advanced myelination were not vulnerable to propofol (Figure 5). This finding is contradictory to the reports from fetal and neonatal NHP studies, in which around 50% apoptotic cells were oligodendrocytes following either propofol or isoflurane exposure [4,9]. Different species-derived oligodendrocytes might have different regulatory signals for oligodendrocyte differentiation and myelination. These differences may underlie the unique susceptibility of NHPs to propofol exposure but not of mice. In addition, since myelination progresses for several weeks in mice, the vulnerable window of oligodendrocytes to apoptosis may not be at P7 in mice. Thus, future studies will include a range of mice ages encompassing the myelination process, especially P20, when myelination peaks. In addition, although propofol did not induce apoptosis in astrocytes, oligodendrocytes, or NSCs in seven-day-old mice, we cannot exclude other toxic effects on these cells. Our previous rat study showed that propofol did not induce the apoptosis of cultured hippocampal astrocytes but decreased the secretion of nine growth factors and cytokines such as brain-derived neurotrophic factor and vascular endothelial growth factor C [24]. These downregulated paracrine factors have been shown to play important roles in neuron survival, learning, and memory [25]. Whether propofol induces such side effect on the mouse hippocampus remains subject to future investigation.

## 5. Conclusions

In this study, we discovered the selective vulnerability of different brain regions, subfields, and different types of brain cells in neonatal mice in response to propofol-induced apoptosis, which will help in better understanding the cellular contribution to propofol-induced developmental neurotoxicity, determining the underlying mechanisms influencing neuronal vulnerability, and further developing targeted therapeutic strategies that specifically protect affected brain areas and neurons.
